# A Career in Manuscripts: Genres and Purposes of a Physician’s Writing in Rome, 1600–1630*

**DOI:** 10.1179/174861811X13009843386639

**Published:** 2011-07

**Authors:** Silvia De Renzi

**Affiliations:** 1 The Open University

**Keywords:** casebooks, early modern physicians, early modern Rome, Giulio Mancini, legal medicine, scribal publication

## Abstract

Following the stellar career of papal physician Giulio Mancini, the article brings into focus learned doctors’ uses of, and relationships with, manu- scripts. Manuscripts were the main outcome of their practice — as letters of consultation to patients and colleagues, as *consilia* of various kinds, including for use in courts of law, and also in the form of key professional tools such as casebooks. Clues found in Mancini’s rich paper-trail shed light on material aspects of his professional writing and on the role that circulating knowledge in manuscript had in creating and sustaining medical networks. The article also argues that even in a domain as shaped by print as early modern medicine, physicians’ use of this medium should not be taken for granted; especially in courtly settings, scribal, as opposed to print, publishing provided them with an effective means of building the social relationships on which their careers depended.

## The manuscript culture of medicine

Reading, collecting, and publishing books in print was central to the self-fashioning of university-educated physicians, and by the early seventeenth century the market in medical publications was flourishing.^1^ Having a book printed served a physician in many ways. It established his credentials as a scholar, teacher, and practitioner beyond the immediate community of his peers: membership of the medical republic was best achieved via the inclusion in the catalogues of international book fairs.

But locally printed defences might also restore a tainted reputation while dedications created or reinforced patronage links. In many ways print made learned physicians. This is hard to dispute, but it is worth considering how this picture originated and whether it can be qualified. The emphasis on the links between medicine and print is partly a result of medical historians’ long-standing preference for academic physi- cians, whose printed output has often provided their best-preserved and most easily available sources. But it is also based on, and reinforces, the assumption that early modern physicians worked with an undisputed hierarchy of media, crowned by print. Both of these historiographical approaches are currently being reconsidered.

Historians are developing a more nuanced understanding of the multiple identities, and sources of recognition, available to early modern physicians, including the profes- sional rather than academic criteria with which patients assessed them. Although elite physicians commonly combined various kinds of employment, the demands experienced by a professor were different from those placed on a town, hospital, or court physician. We see this in the different genres of medical writing they would mainly produce: academic commentaries, tracts on specific doctrinal points, collections of cases, or the multiple genres in which they gave medical advice, from regimina — sets of rules to preserve health tailored to a patient’s unique temperament — to a variety of other pieces of advice, or *consilia*. Each of these was characterized by centuries-old conventions including expected forms of circulation. Evidence that print could be just one of the options to build one’s profile as a respected physician comes from Rome, where, although printers had been quick to take advantage of the rising demand for medical knowledge,^2^ of the eighteen physicians who served as *protomedico* — head of the college of physicians — between 1600 and 1630, only four published in print. Evidently, the majority established their eminence otherwise. It is also revealing that the entries in a contemporary compilation of illustrious men, including physicians, list manuscripts and printed works together, a clear indication that in Rome the former were by no means regarded as failed publications.^3^

Thanks to the work of literary scholars, a rich picture of the etiquette and purposes of scribal publication is now informing historical research more broadly. The fluid interactions, rather than the hierarchy, between media — print, manuscript, even orality — have been brought into focus, including how the choice of medium depended on discipline-specific regimes and expectations.^4^ In my analysis of early modern physicians’ uses of media I draw on this kind of reassessment, but by rethink- ing their participation in manuscript culture I also look afresh at their professional writing. Taking notes at the bedside, drafting advice to patients and colleagues, jotting down recipes, and, especially on the Continent, giving written medico-legal advice: early modern physicians wrote a great deal, and manuscripts, here in the sense of both the intermediate and the final products of writing for the profession, were a constant output of their activities. Sources for recapturing physicians’ writing as a process are rare and made problematic by the often complex afterlife of their archives, but they allow historians of medicine to contribute to broader research into the technologies of writing for practical or professional purposes.^5^ Exploring the materiality of medical writing can also fruitfully intersect with current developments in the history of medicine. For their daily work, physicians would use well-established professional tools such as casebooks where they would record encounters with their patients. These have provided historians with a key source for recapturing patients’ views and patient–practitioner relationships, and research is now looking at the material processes and social interactions leading to the compilation of casebooks.^6^

Recent work has shown that early modern physicians were increasingly keen on collecting and sharing observations and medical histories, an activity bound up with important epistemological shifts such as the rising status of first-hand accounts of individual cases. While much of the evidence has come from printed collections, exploring the production of the records that would be selected for print can reveal the different kinds of practice, including writing, that nourished new forms of medical learning.^7^

An excellent figure through whom to recapture physicians’ multifaceted relation- ships with writing and manuscript culture is Giulio Mancini, the doctor at the centre of this article. By any standard he belonged to the elite of the profession: in 1623, he became doctor to Pope Urban VIII, the apex of a physician’s career at the centre of the Catholic world. However, this was no reward for academic glory or the publication of dense commentaries on canonical texts. Mancini never had his works printed, partly perhaps the result of his decision to remain an important but behind-the-scenes participant in the doctrinal disputes that shook the Roman medical community. Chiefly a practitioner, to reach his position, reputation, and wealth, he resorted to other means which in Rome were effective and familiar to his colleagues. His determination in mobilizing his expertise to climb the social ladder was excep- tional, but the range of medical genres and advisory roles he took on was common. Mancini is unusual in the breadth of his non-medical interests. The *Considerazioni sulla Pittura* — his best-known tract which had wide manuscript circulation but was printed only in the mid-twentieth century — gave a new twist to humanist physicians’ interest in history and literature and has provided art historians with a key source for art in baroque Rome.^8^ He also wrote on such varied topics as the sources of honour, the pedagogical merits of drawing and dance, and the origin of money.^9^ While apply- ing to all these subjects the Aristotelian perspective he had learnt in Padua, he showed the wide-ranging competence that a physician could offer to patrons and patients. More than just idiosyncratic, his uncommon breadth of interests demonstrates the different services to which early modern physicians would put their education.

Handwriting, then, not printing, permeated Mancini’s private and professional life, as shown in the abundant paper-trail he left in the Vatican Library, the Biblioteca Comunale of Siena, and the family archive, including regular correspondence with his relatives.^10^ Using this rich documentation, I will reconstruct here a physician’s writing both in his day-to-day professional duties and in his broader effort to establish a reputation and win patronage. My analysis is chronological so as to recapture the full range of Mancini’s dealing with manuscripts at each stage of his progression from student to papal physician.

## From student to practitioner

Leaving Padua in 1585, after six years of medical studies, Mancini could be proud of the social and intellectual experience he had gained by skilful networking.^11^ He had built a close relationship with the illustrious professor Girolamo Mercuriale and, as a token of esteem, oversaw the printing of Mercuriale’s *De decoratione*, Mancini’s first and last encounter with the press.^12^ He had also been admitted into the renowned library of the scholar Gian Vincenzo Pinelli, whose collection of manuscripts included, among much else, political tracts and university lecture notes.^13^ Mancini duly con- tributed to it by sending from Bologna, where he lived for a few months, the notes of local professors and helping Pinelli locate various manuscripts, including ancient copies of Latin authors.^14^ Still young, Mancini was actively participating in the key cultural practice of providing manuscripts as a lubricant for intellectual and social relations.^15^ Since his early education he had been exposed to the scribal culture still thriving in the literary and academic scene of Siena.^16^ In Padua, as his intellec- tual horizons expanded, so did his manuscript practice. As a token of gratitude, he would send his former teachers the notes of lectures he attended in his *peregrinatio academica*.^17^ As a conscientious student, Mancini was also building his own collec- tion of lecture notes, which he commissioned and diligently took home.^18^ For those who could afford them, the lecture notes of renowned teachers would double as professional tools and cultural trophies. We know very little about Mancini’s library, but when, back in Siena, he started to practise and enjoyed (albeit briefly) the perks of academic life,^19^ the newly acquired notes of Paduan professors must have sat next to notes dating back to his early education in Siena and it is tempting to imagine him preparing his own anatomical lectures while consulting those volumes.^20^

Writing was integral to his medical practice too, as shown in a manuscript entitled ‘Consilii varii et medicamenti’.^21^ Writing *consilia* had been the bread and butter of a learned physician’s practice since the late Middle Ages. Usually sent to a patient or colleague who had asked for advice on an individual case, medieval *consilia* were steeped in the doctrine of which they were illustrations. Therapy was included but an account of the progress of the disease was not.^22^ As recent research has shown, new genres of medical writing emerged in the sixteenth century, from *curationes* to *observationes,* signalling an important epistemological shift whereby narratives prevailed over doctrinal discussion (see Pomata). These new kinds of writing, how- ever, did not remove the demand for consulting through *consilia* which physicians, in a variety of capacities, were still writing in the seventeenth century.^23^ This is what Mancini meant by ‘consilii’: his volume includes drafts of the letters he sent other physicians and patients, providing his views on the causes of disease and advice on therapeutics. Take for example the two-page discussion of a case of melancholy or mania and the recommended therapy, which was introduced with the expression: ‘per ubbidir i comandamenti del Signor Antonio Pino, mio singolarissimo padrone’.^24^

As for any other early modern physician, providing *consilia* was an important social tool for Mancini, and he used his book to draft and revise them before copying and sending them off. But in the volume, these drafts are mixed up with entries which seem to be simple records of his daily medical encounters and which he probably understood as ‘medicamenti’.

These are introduced by a precise identification of the patient, as in ‘Nicolao dal Sorno 27 anni’, or a generic indication: ‘puella annorum 15’, or ‘donna contadina’.^25^

The length of the entries varies and some are very short, as where Mancini sums up the case of a sixteen-year-old boy in just eleven lines. He had a weak pulse, a confused mind, and pain all over the body; Mancini guessed malignant fever, and after spots had appeared he ordered a syrup and an ointment. Such records were clearly for Mancini’s own use.

The volume, then, was a rough book which Mancini used as he went about his medical practice — there is some doodling, too. It is a folio, so could not be taken along when Mancini went to see bedridden patients, although the occasional ‘venne’ indicates that he also saw patients at his home. Entries must have followed the order of the medical encounters or of the requests for *consilia* but no further chronological arrangement (e.g. by week or month) was imposed and a simple line separates each one. Some cases, however, do include a date, usually the time of the visit or the onset of a disease. On fol. 45, for example, an eight-line record introduced with ‘pro D. Ioanne Columbino’ begins with the date of the onset of the ailment (‘2 Januarii’). A brief description of the symptoms is followed by the comment that on the second day (of the disease) Mancini was summoned. So the expression ‘hier l’altro’ scribbled close to the date reveals that he was entering his notes on the day of the visit. How- ever, many entries include an account of the progress of the disease, and this suggests that Mancini would take rough notes over a number of days and then reorganize them by case before entering them in the book. So this could already be one step removed from the medical encounter. A competing procedure was also in place, by which Mancini would allocate pages to patients before writing on them. He would allow roughly a half-page each. On fol. 106^v^, for example, names of patients are crossed out at the top and middle of the page to make space for a longer discussion of how to treat a broken skull which starts on fol. 105^v^ and carries on to fol. 107. An unexpectedly detailed doctrinal disquisition, perhaps connected to his teaching, disturbed the prior arrangement.

Mancini’s casebook is akin to those of other learned physicians, but some of these were at some point edited — in preparation for publication that may or may not have happened — while Mancini’s book remained untouched probably until it entered the Biblioteca Comunale in Siena, when pagination was added.^26^ The hybridity of the volume — rough book and recording log — provides glimpses of the more material arrangement that sustained professional writing and illustrates the making of a crucial professional tool. How to interrogate a patient was part of medical training, but organizing a casebook was probably learnt on the job and we may wonder if recording practices from outside the profession provided models too. Because patients might return, retrieval of information was important, but no finding-aides or indexes were added, and given how crammed the pages are, locating cases must have been easier for patients of higher status who were introduced by a full name. However, the brief report of the post mortem appended at the end of the entry for a patient (though probably in a different handwriting) is evidence that Mancini would go through his casebook: findings at autopsy were becoming integral to the consideration of a case and provided another reason to revisit it. An important professional tool, the book followed Mancini to Rome and he used it in his later life.

## Professional writing: breadth, techniques, audiences

So far I have looked at how a young and ambitious physician would relate to differ- ent kinds of manuscripts: as a medium of medical education, as sought-after cultural relics that would mediate social interactions, and as the outcome of professional writing. I now move to explore how manuscripts shaped Mancini’s blossoming career in Rome. I shall continue examining how techniques of writing helped him in his professional duties, but two other issues will also come into focus: the shared authorship of his works and their circulation among a varied audience.

Something in Siena went wrong for Mancini and, after a spell in Viterbo, in 1592 he settled in Rome with a position at the prestigious hospital of Santo Spirito. Rome’s high density of wealthy households attracted young physicians out to make their fortunes. Letters to his cousin Claudio and his brother Deifebo bear witness to Mancini’s growing professional reputation, impatience with hospital chores, and consummate financial skill, including in the art market where he invested the profits from his lucrative practice: but he seems never to have been tempted by academia again. His interest in manuscripts did not wane and in 1596 he asked Claudio to check if his old friend Rosso, a bookseller in Siena, might have books ‘scritti a mano’;^27^ he was also interested in ‘libri di cartapecora’ and if these were in good condition, he advised Claudio to buy them at one giulio a pound.^28^ What Mancini wanted with manuscripts bought by weight is hard to say: vellum could perhaps be scraped and reused for writing. Procuring manuscripts, however, was an efficient way to ingratiate himself with patrons such as the Commendatore of Santo Spirito. This influential prelate responsible for the administration of the hospital was in the 1590s a fellow-countryman who doubled as the bishop of Montepulciano. Inter- ested in a local saint, he must have inquired about what was available on her and Mancini mediated between him and an acquaintance, who was in a position to lend a manuscript about the saint: by way of a deposit, the man was offered a manuscript ‘di non minor pregio’.^29^ As in Padua, so too in the religious milieu of Rome, dealing with manuscripts, old and new, was intellectually and socially rewarding. However, Mancini was also eager to be reunited with manuscripts of a different kind, medical tracts he had left behind and now needed for his profession. So he requested that his cousin send over two he had written (on petechial spots and on crisis) as well as an ‘Avicenna’ with boarded covers.^30^

These works would help in his daily practice, an important component of which was writing *regimina* and *consilia* with therapeutic instructions for individual patients. To show he was an experienced practitioner, Mancini would often mention cases similar to the one at hand, from his hospital and private practice as well as from historical sources. Considering the mutism of the Prince of Parma, Mancini recalled a case discussed by Tacitus, and that of a boy at Santo Spirito who, dumb since his childhood, had gained the ability to speak.^31^ Unlike for Siena, no book recording Mancini’s medical encounters in Rome is extant, and we can only speculate about how, when writing about a Roman nobleman’s loss of voice, he would locate details of such other cases as that of ‘an apothecary in Trastevere’ and ‘a woman living near Santa Marta’, to whom he compared his patient’s condition.^32^ In one instance, however, we can positively trace a case to the casebook from Siena. Writing for a patient who suffered from painful urination, Mancini recalled that thirty years before he had observed something similar in Ventidio Beccafumo, the patient I mentioned above, whose notes had included the result of a post mortem.^33^ Moving between old and new cases, Mancini used his casebook as a repository of observations and therapies guiding his profession.

Like many of his colleagues, Mancini combined medical practice with acting as a sought-after expert witness in the Roman tribunals. Continental legal procedure stipulated that physicians could give their expert testimony at court by submitting medico-legal *consilia* to be included in the trial record. So here was another reason to put medical knowledge on paper, tailoring broad doctrine to the specifics of a case. Once again Mancini allows us to glimpse the material aspects of this laborious process. In 1609, he was involved in a high-profile case of suspected poisoning. A neat copy of Mancini’s testimony, probably produced by a scribe, is where we expect it, in the several-hundred-page legal folder.^34^ However, the first pages of his *consilium*, in the same handwriting, also appear in a bulky and chaotic folder comprising an array of Mancini’s papers now in Siena.^35^ The copy includes additions in Mancini’s writing and after a few pages trails off into untidy notes also in his hand, revealing that this was an intermediate stage in the production of the testimony. Copies of legal documents follow, for example, other physicians’ expert testimonies, and it may surprise us to find what we would regard as official records included among private papers. In the all-written procedure of continental tribunals, legal documents were made available to the parties’ lawyers in copies routinely produced by notaries,^36^ and would be passed on to expert witnesses, ending up among their working (private) papers.

So, in possession of legal documents, how did Mancini produce his *consilium*? Establishing the facts was as critical as it was contentious.^37^ The first page reports the events leading to the suspicious death: they are listed in a column on the left-hand side of the paper, with key words identified by a number. On the right-hand side a numbered list connects the corresponding word to the name of the witness who provided the information. Thus, alleged facts and their sources are correlated and available at a glance, making it easier to draw on, or challenge, them. Producing a medico-legal *consilium* required navigating through a thick legal dossier, a daunting task made feasible by simple paper technology and synoptic aids. As we know from studies of commonplace books and indexes, Renaissance readers had various tech- niques for taking notes, excerpting, arranging and making retrievable increasingly abundant information, but the layout of Mancini’s testimony takes us to the still little-investigated level of the strategies adopted to discharge professional duties.^38^

By the 1610s, stimulated by Rome’s courtly culture, Mancini was expanding the areas on which he felt competent to provide written advice. He entered two heated controversies on matters of precedence in which, as a physician, he had a stake: one involved the Commendatore of Santo Spirito.^39^ But he was also increasingly writing ‘scritture’ on topics that would stir his (actual and potential) patients’ curiosity. So in 1611 he started a tract on preserving health (‘de sanitate tuenda’) which, although it had probably been commissioned by a patient, certainly appealed to many, and one on diseases of the soul.^40^ Over the next ten years he ventured outside his profes- sional turf, producing and revising tracts on such varied topics as love, the sources of honour, and which variety of Italian was best suited for the curia. A physician looking for potential clients, he was fashioning himself as a generally accomplished advisor on contemporary preoccupations.

Writing, however, was demanding — a letter portrays Mancini at work in the middle of the night^41^ — and protracted, especially because, probably unsure of his style, he depended on the comments of family and friends back in Siena. A pattern was quickly established by which, enclosed in letters to his brother, copies of his drafts would travel to Siena, from where Mancini eagerly awaited feedback. In February 1612 when working on ‘de sanitate tuenda’, Mancini said that he was expecting Signor Paride’s judgement which should be sent by courier.^42^ Professor of *medicina theorica* in Siena, Paris Biringucci remained a source of advice for years to come. How work progressed amid the responses of various readers emerges in a letter in which Mancini took stock of his activities.^43^ First he solicited his brother’s and Biringucci’s advice as to whether the medico-legal *consilia* he had sent (probably including the one about the alleged poisoning) were worth publishing; then he listed his recent writings, a compendium on astrology and a tract on the ‘critical days’ — a key issue for physicians’ prognosis — and also various ‘discorsi civili’ which, he explained, he had composed for his patrons. The influential Cardinal Luigi Capponi, also a patient, had liked one of them and yet Mancini would appreciate his brother’s comments. Finally, he boasted that another medico-legal *consilium* had been much appreciated by the judge and many physicians had requested copies. The letter illus- trates the range of audiences Mancini reached in his different capacities at any one time, from patrons to whom he submitted work in progress to colleagues who followed with interest (and probably also a wary eye) his handling of a legal case.

Not only did the traffic of copies between Rome and Siena make writing a shared exercise; Mancini was also willing to hand over authorship. In June 1612, considering again the publication of the well-received medico-legal *consilium*, he indicated that difficulties might be raised in Rome and the work could preferably come out in Siena under a pseudonym. It is unclear what the obstacles may have been and, although publishing under other people’s names was not unusual, it is hard to believe that the trick would have worked in relatively small circles.^44^ Print publication was clearly in Mancini’s mind, but nothing came of these projects: the production and circulation of manuscript copies was his main concern.

## The changing purposes of copies

In sending copies of his tracts to patrons, Mancini certainly shared in a culture of gift exchange, but copies did not have to be expensively produced. What mattered was to have patrons mention, or materially circulate, his works in increasingly influential milieux, which meant closer to the papal court. In 1620 when busy writing his ‘Considerazioni sulla pittura’, he explained that Cardinals Capponi and Barberini had seen and liked it and the former would show it ‘più alto’.^45^ Scholars have com- mented on the vertical circulation of manuscripts, and competition for the attention of a high-status audience was fierce in seventeenth-century Rome.^46^ A constant flow of literary, natural philosophical, and erudite works would be presented by clients in search of patronage while, in the highly charged environment of the 1610s, political manuscripts could steal the scene. Mancini himself shows the wide circulation of accusations, apologies, and responses through which religious and political contro- versies were fought out. In 1617 he wrote that a defence by the controversial Angelo Badoer — who had recently been charged with espionage and banned from Venice — was circulating and that he would have had it copied — but for some reason had not.^47^

Keeping one’s patrons interested was hard, but Mancini’s audience also included a more horizontal network of peers, including outside Rome. For example he made sure that his brother Deifebo produced and forwarded copies of his writings to a gentleman from Cremona who persistently requested them, ‘acciò dia gusto a questo gentilhuomo’.^48^ Pleasing the gentleman did not bring any immediate gain but, in addition to complying with the etiquette of the gift, Mancini must have been flattered: one of his tracts on precedence became the blueprint for the gentleman’s own contri- bution in a similar controversy. Other eager readers to whom copies were regularly given included a doctor from Viterbo, probably met during Mancini’s time there. However, there is evidence that Mancini and his brother also fretted about who should have access — and be allowed to make copies — of his work. Sometimes Mancini imposed a strict policy: while a work could be shown, it should not be copied; perhaps plagiarism could become an issue after all.^49^

Historians have recently highlighted that sharing cases with colleagues became increasingly important to early modern physicians, and this must have included send- ing transcriptions from one’s notes and records in letters (see Pomata). It is hard to establish whether Mancini took part in this new game, but a volume associated with him illustrates alternative practices of copying and circulating a physician’s work and the various purposes of making medical manuscripts ‘public’ within certain net- works. The volume, entitled ‘Practicae medicinae vectigal’ (A tribute to the practice of medicine) is an assemblage of twenty-eight tracts by various authors; written at different times, they had circulated in other copies before being copied here.^50^ The words ‘Julii Mancini’ on the title-page link the volume to our physician, though he could be either the person to whose practice of medicine the book was a tribute, or the owner of the manuscript. Most of the tracts have a strong practical nature. Lists of drugs are included with *consilia* and *regimina* for individual patients, responses to specific medical controversies, methods of cure and treatments carried out by named physicians; some have a more academic origin. A final index indicates that the collection was intended as a repository of medical information; here, however, I am mostly interested in its production.

The colophons of some tracts reveal the copyist as Sebastiano Vannini, a young doctor who after a degree in Siena spent time with Mancini in Rome, including as a copyist for the ‘Considerazioni’.^51^ It is reasonable to assume that Vannini copied all of the tracts between 1617 and 1619. Six tracts are the work of Pirro Bizzarrini, Vannini’s professor in Siena and a member of the College of Physicians. Bizzarrini also owned tracts which he lent Vannini to be copied. Other Siena professors figure as authors in the collection, from Cipriano Casolani — here represented with ‘Curationes febrium putridarum’ (Cures for putrid fevers) and a *consilium* — to Paris Biringucci, Mancini’s advisor and here the co-author of a *consilium*. A model for this assemblage is medieval collections of medical tracts, an important profes- sional tool with a strong practical component.^52^ In the age of print, the assemblage acquired other meanings and can be understood within the practice common among literati of putting together manuscript works which, by reflecting their common inter- est, strengthened the relationship of a closely linked group of people.^53^ The collection was then in part the token of gratitude of a student willing to circulate the works of his teachers and the manuscripts they possessed. Coming to Rome, Vannini may have decided that the collection could double as an appropriate gift to his new mentor Mancini, who, as the young doctor knew, was eager to cultivate his Tuscan identity.

The assemblage also reveals how a widespread practice of exchanging and copying manuscripts would circulate medical knowledge near and far. Take the tract ‘Meth- odus compendiosa curationi morborum inserviens’ (A compendious method for the cure of diseases) which Vannini copied from an exemplar borrowed from Giovanni Andrea Selvagno Cavensi (or Cevensi), Mancini’s assistant at Santo Spirito. Origi- nally from Piedmont, Selvagno Cavensi claimed that the text had been dictated to him by Orazio Augenio, professor at the University of Turin. On leaving Piedmont to make his fortune, Selvagno Cavensi must have taken the notes with him and was then happy for Vannini to copy and add them to the collection of manuscripts for their common mentor. We know little about the day-to-day life of practitioners in early modern hospitals, but, as in more academic environments, making copies of medical works mediated the relationships between junior and senior physicians. Furthermore, Vannini’s borrowing from Selvagno Cavensi, facilitated by their association with Mancini, confirms the important role of Santo Spirito as a hub of medical communi- cation, presumably both verbal and through the exchange of manuscripts. Unsurpris- ingly, Mancini’s *regimen* for the Commendatore of Santo Spirito is included in the collection.

Making copies was obviously meant to circulate one’s works, but Mancini also allows us to appreciate the more mundane purpose of counteracting frequent mate- rial damage. This could happen in two ways: however well disposed, patrons could be careless and, as Mancini realized to his chagrin, lose the manuscripts they had received.^54^ But he was also concerned about physical deterioration — ‘andare a male’ is the recurrent expression — that could affect his own copies.^55^ In July 1621 he announced that a copy was ‘andata a male’ — at this point he was probably living in an apartment in Santo Spirito which was located in a damp area of Rome — and in trepidation he asked if Deifebo had another.^56^ An upset Mancini wrote a few weeks later that he was surprised Deifebo took so little care of his ‘scritture’. He reminded Deifebo to keep the copies he received and make sure they were not damaged.^57^ His disappointment did not affect the conviction that the house in Siena should become a repository of his writings: copies that had been lent should be returned there and kept with his other ‘cose’.^58^ An avid collector, Mancini would regularly send the art he bought in Rome to Deifebo, who, at the same time, was building a family archive.^59^ Doubling as a store for objects and papers, the family house was the most appropriate place to collect Mancini’s manuscripts; in the absence of print publication, it was also from here that copies could be produced on demand.

Between his arrival in Rome in 1592 and the early 1620s Mancini wrote exten- sively and with a range of different purposes, but regardless of the specific aim or content, he followed a regular sequence. From Rome his manuscripts took the road to Siena, to be read, commented upon, and improved. Writing was much more shared than private. If printing was considered, it never happened; but disseminating manu- script copies of what was in many ways work in progress could be equally effective in achieving Mancini’s chief goal of boosting his profile as a physician and a courtier. Entrusting his brother with the preservation of his professional archive was part of the strategy. Things changed, however, with his appointment as papal physician.

Mancini probably negotiated to keep a separate residence and had permission to practise, but his daily schedule was redrawn and he now had a demanding reader to entertain. In December 1623 he sent Deifebo the draft of a ‘discorso’ on a recent flood which the Pope, to whom a fair copy had been presented, had enjoyed.^60^ Having read and taken pleasure in a number of his works, the Pope provided Mancini with a paid scribe to recopy all his writings.^61^ Accordingly, Mancini asked Deifebo to send over the manuscripts accumulated over the years in Siena — together with new bedding to be used at court. Making copies acquired a new meaning as the disparate output of a busy, but not academic physician, coalesced as a gift to the Pope. Late in life — he was now sixty-four — Mancini had achieved publication, and that it was scribal did not matter. Probably in the process of taking stock of his output, he compiled a lengthy list of over fifty titles.^62^

Four volumes in the Barberiniani Latini collection in the Vatican Library may represent a stage in the production of Mancini’s corpus.^63^ Although they are not a presentation copy — corrections show that he was revising them — the volumes remained in the library of Urban VIII’s family and, taken together, they convey the breadth of a physician’s advisory role — the professional feature Mancini was eager to project. Scholars have mainly focused on the text of the ‘Considerazioni’ included here, but Mancini was inclusive: the whole range of his writings — non-medical works, medico-legal and medical *consilia* — is represented. The volumes are hetero- geneous in content, with the exception of MS Barb. Lat. 4317, which includes only *consilia* and *regimina* — and they are all from his time in Rome: his practice there was meant to speak of his skills. The presence of Mancini’s medico-legal *consilia* demonstrates the high regard in which physicians — and their patrons — held this side of medical expertise.^64^ A comparison between the copy of a *consilium* Mancini had submitted to the court and the one he had copied for the pope reveals that he had handed the copyist the whole set of notes he had accumulated while dealing with the case, including the legal documents at his disposal and working notes with disparaging comments and gossip about the case.^65^ Very likely these would have been eliminated in a subsequent editing, but this never took place: striving to put together the corpus that would represent a physician’s life to the pope, Mancini remained trapped in ongoing revisions and a 1627 letter shows him busily writing in bed and surrounded by a lamp, paper, and an inkwell, while still improving the tract on honour.^66^ He died in 1630.

## Conclusion

Occasionally Mancini thought about printing his works: he obviously appreciated the advantages this could bring. However, it did not happen and we may wonder how strongly he really pursued this aim; my conclusion, rather, is that he came to see scribal publication as an equally effective way of climbing the professional and social ladder in seventeenth-century Rome. His stellar career proved him correct and this article has demonstrated the resources and circumstances that made his the right choice. On the one hand, from his student days Mancini shared in the multifarious aspects of a culture in which commerce in manuscripts — locating, providing, and copying them — was an effective social lubricant. On the other hand, opting out of academia and coming to Rome, where courtly codes dominated and success was measured by proximity to power, made circulating one’s works to targeted audiences the best way to show the credentials of a skilled practitioner who could double as an adviser on an extraordinarily wide range of topics; printing was not necessary to reach the indispensable patrons. The broad lesson to draw is that even within a domain as informed by print as early modern learned medicine, we should not take the use of this medium for granted; there were still ample reasons that made other media, and engaging with the narrower and more targeted audiences they afforded, a perfectly sensible and effective option. The choice made by physicians was based on local, social, and professional considerations, including the genres in which they would write, since genres imply audiences and project identities. The pressure to print publish was higher for university professors and I do not want to underestimate the service it provided to non-academic physicians, too, but archival investigation may reveal that both skilfully moved between alternative and complementary media.

The case of Mancini also allows us to reconsider the material aspects of writing in a learned physician’s daily activity. His casebook has revealed the most basic stage of writing, which took place soon after, though not during, a visit. Recording the details of a patient made them available for subsequent retrieval, something that Mancini did in his later practice, proving that casebooks, to our eyes rather messy objects, worked as a source of guidance. The lack of aids that would help locate cases was no obstacle, but material clues in his medico-legal *consilia* have shed light on the specific techniques with which an expert witness could extract from numerous testimonies the ‘fact’ on which his competence was required. Research into the paper technology supporting physicians’ work can add an important dimension to our understanding of the materiality of Renaissance learning.

Exploring how Mancini circulated his work has highlighted the various audiences that manuscripts could reach, from patrons to more ordinary readers and the peer community, whose pressure is apparent in Mancini’s lack of confidence and dependence on suggestions from loyal friends among physicians and literati. Their negotiations, however, also signal the fluid boundaries of authorship. More broadly, Mancini’s association with the production of the ‘Vectigal’ has revealed how, even in the ‘age of print’, scribal practices — lending, copying, assembling manuscripts — worked to create and sustain the diverse relations — among peers and between junior and senior colleagues — which constituted a physician’s professional network. The scribal medium presented constraints as well as opportunities: in the absence of print, preserving copies was crucial and Mancini’s concerns also provide insight into the question of how professional papers fit into family archives. A solution to the fragility of the medium came from within scribal culture as the pope offered a copyist. Historians have traditionally regarded *consilia* for patients and tribunals, not to mention tracts for patrons, as the uninspiring side of early modern medicine, but they created Mancini’s success; now rescued and recopied, they could be turned into a collection fit for the pope. Reaching out to a wide and scattered audience through print could even appear no great achievement if the alternative was to offer a selection of one’s work to God’s representative on earth.

## References

[1] Maclean Ian, Logic, Signs and Nature in the Renaissance: The Case of Learned Medicine (Cambridge: Cambridge University Press, 2002), pp. 36–67

[2] Pinon Laurent, ‘La Culture scientifique à Rome au miroir des livres (1527–1650): apports et limites de l’approche bibliographique’, in *Rome et la science moderne entre Renaissance et Lumières*, ed. by Romano Antonella, ed (Rome: École française de Rome, 2008), pp. 173–206

[3] Allacci Leone, Apes urbanae (Rome: Ludovicus Grignanus, 1633).

[4] For examples and bibliography on the new approach, see Brian Richardson, *Manuscript Culture in Renaissance Italy* (Cambridge: Cambridge University Press, 2009); Silvia De Renzi, ‘Writing and Talking of Exotic Animals’, in *Books and the Sciences in History*, ed. by Marina Frasca-Spada and Nick Jardine (Cambridge: Cambridge University Press, 2000), pp. 151–67; Mario Biagioli, *Galileo’s Instruments of Credit: Telescopes, Images, Secrecy* (Chicago: University of Chicago Press, 2006).

[5] Domenico Bertoloni Meli, ‘The Archive and *Consulti* of Marcello Malpighi: Some Preliminary Reflections’, in *Archives of the Scientific Revolution: The Formation and Exchange of Ideas in Seventeenth-Century Europe*, ed. by Hunter Michael, ed (Woodbridge: Boydell, 1998), pp. 109–20 See Laurie Nussdorfer, *Brokers of Public Trust: Notaries in Early Modern Rome* (Baltimore: Johns Hopkins University Press, 2009) for exploration of legal writing

[6] MacDonald Michael, Mystical Bedlam: Madness, Anxiety, and Healing in Seventeenth-Century England (Cambridge: Cambridge University Press, 1981); Barbara Duden, *The Woman beneath the Skin: A Doctor’s Patients in Eighteenth-Century Germany* (Cambridge, MA: Harvard University Press, 1991); Donatella Barto- lini, *Medici e comunità: esempi dalla terraferma veneta dei secoli XVI e XVII* (Venice: Deputazione Editrice, 2006), pp. 199–209; see also the project at 〈http://www.hps.cam.ac.uk/casebooks/〉 [accessed 20 October 2010]. On the material evidence of a casebook: Brian Nance, *Turquet de Mayerne as Baroque Physician: The Art of Medical Portraiture* (Amsterdam: Rodopi, 2001), Chapter 2.

[7] Pomata Gianna, ‘Sharing Cases: The *Observationes* in Early Modern Medicine’, Early Science and Medicine, 15 (2010), 193–236, where she briefly engages with manuscript collections of *curationes* 20695394 10.1163/157338210x493932

[8] Mancini Giulio, Considerazioni sulla pittura, ed. by Marucchi Adriana Salerno Luigi, ed, 2 vols (Rome: Accademia Nazionale dei Lincei, 1956–57). On physicians’ erudition, see Nancy G. Siraisi, *History, Medicine, and the Traditions of Renaissance Learning* (Ann Arbor: University of Michigan Press, 2007)

[9] On Mancini’s political tracts, see Antonio Menniti Ippolito, ‘Nella Corte di Roma, o per dir meglio | nel pubblico spedal della speranza’: note per una lettura dall’interno della curia romana seicentesca’, Annali di storia moderna e contemporanea, 4 (1998), 221–43

[10] The family archive is in the Archivio della Società di Esecutori di Pie Disposizioni in Siena (hereafter ASEPD).

[11] For biographical information, see Michele Maccherini, ‘Caravaggio nel carteggio familiare di Giulio Mancini’, Prospettiva, 86 (1997), 71–92; Silvia De Renzi and Donatella Sparti, ‘Giulio Mancini’, in *Dizionario biografico degli Italiani*, 68 (Rome: Istituto dell’Enciclopedia Italiana, 2007), 500–09, to which I refer for further bibliography; Silvia De Renzi, ‘Medical Competence, Anatomy and the Polity in Seventeenth-Century Rome’, *Renaissance Studies*, 21 (2007), 551–67.

[12] De decoratione liber […] ex Hieronimi Mercurialis […] explicationibus: a Iulio Mancino exceptus primum, *& in capita redactus* (Venice: apud Paulum Meietum, 1585).

[13] Nuovo Angela, ‘The Creation and Dispersal of the Library of Gian Vincenzo Pinelli’, in *Books on the Move: Tracking Copies through Collections and the Book Trade*, ed. by Myers Robin Harris Michael Mandelbrote Giles, ed (New Castle: Oak Knoll Press, 2007), pp. 39–67, and the essay by Nuovo in this volume

[14] ASEPD, C XIX 166, Pinelli to Mancini, fols 263–64, 301, 304, 305.

[15] For the Italian context, see Richardson, pp. 1–58.

[16] Bartalini Roberto, ‘Siena medicea: l’Accademia di Ippolito Agostini’, Annali della Scuola Normale Superiore di Pisa, Classe di Lettere e Filosofia, 3rd ser., 25 (1995), 1475–1530

[17] ASEPD, C XIX 166, fol. 286.

[18] ASEPD, C XIX 166, fol. 135^v^.

[19] On Mancini’s chair in anatomy, see Giovanni Cascio Pratilli, *L’Università e il Principe: gli studi di Siena e di* Pisa tra Rinascimento e Controriforma (Florence: Olschki, 1975).

[20] Manuscripts in the Biblioteca Comunale di Siena (hereafter BCS) with a common shelf mark (C IX) have been linked to Mancini. In addition to internal evidence, the strongest link with Mancini is in a nineteenth-century document in the family archive (ASEPD, C XIX 167, fols. 160–67) where most of them are listed. This suggests that they were part of the family archive before being moved to the Biblioteca. They include notes of a profes- sor of philosophy in Siena (BCS, C IX 9), lectures by Fabrici D’Acquapendente and Mercuriale (BCS, C IX 27 and 22), and notes arranged under the headings with which anatomy was taught (BCS, C IX 7). I thank Drs Katia Cestelli, Milena Pagni, and Rossella De Pierro for their help in this matter.

[21] BCS, C IX 4 The response to a question about the likelihood of plague, dated 1587, is the only explicit clue as to the date of composition.

[22] Crisciani Chiara, ‘L’individuale nella medicina tra medioevo e umanesimo: i ‘consilia’’, in *Umanesimo e medicina: il problema dell’‘individuale’*, ed. by Cardini Roberto Regoliosi Mariangela, ed (Rome: Bulzoni, 1996), pp. 1–32

[23] Chiara Crisciani, ‘*Consilia*, responsi, consulti: i pareri del medico tra insegnamento e professione’, in *Consilium: teorie e pratiche del consigliare nella cultura medievale*, ed. by Casagrande Carla Crisciani Chiara Vecchio Silvana, ed (Florence: Sismel-Edizioni del Galluzzo, 2004), pp. 259–79

[24] BCS, C IX 4, fol. 147^v^.

[25] BCS, C IX 4, fols. 24^v^, 4, 19^v^.

[26] As Nance shows, in his old age de Mayerne edited his casebook for publication, as did the Dr Chiavenna discussed by Bartolini.

[27] 24 February 1596, transcribed in Michele Maccherini, ‘Caravaggio e i caravaggeschi nel carteggio familiare di Giulio Mancini’ (unpublished doctoral thesis, Università degli Studi di Roma La Sapienza, 1990–93), pp. 159–60.

[28] 13 January 1596, Maccherini, ‘Caravaggio e i caravaggeschi’, p. 159.

[29] 24 February 1596, Maccherini, ‘Caravaggio e i caravaggeschi’, pp. 159–60.

[30] 18 December and 5 November 1593, Maccherini, ‘Caravaggio e i caravaggeschi’, pp. 157–58.

[31] Biblioteca Apostolica Vaticana (hereafter BAV), MS Barb. Lat. 4317, fol. 10. See below on the origin of this manuscript.

[32] MS Barb. Lat. 4317, fol. 54.

[33] MS Barb. Lat. 4317, fol. 34^v^.

[34] Archivio di Stato di Roma, Tribunale del Governatore, Processi sec. XVII, b. 81, fols 751–59^v^.

[35] BCS, C IX 3; the content of this folder was at some point divided into smaller, numbered folders; papers related to the case are in folders 92–102

[36] Pompeo Augusto, ‘Il tribunale criminale del Governatore di Roma nella seconda metà del XVI secolo’, in *Sisto V*, i. *Roma e il Lazio*, ed. by Marcello Fagiolo and Maria Luisa Madonna (Rome: Istituto Poligrafico e Zecca dello Stato, 1992), pp. 97–110

[37] Renzi Silvia De, ‘La natura in tribunale: conoscenze e pratiche medico-legali a Roma nel XVII secolo’, Quaderni storici, 108 (2001), 799–822 18841616

[38] Blair Ann, ‘Reading Strategies for Coping with Information Overload, ca. 1550–1700’, Journal of the History of Ideas, 64 (2003), 11–28 12841169

[39] Copies of both are now in BAV, MS Barb. Lat. 4315.

[40] A description of ‘de sanitate tuenda’ is in a letter of 16 April 1611: ASEPD, C XIX 168, fol. 556^r–v^; on the ‘de morbis animi’, ibid., fol. 568.

[41] 10 March 1612: ASEPD, C XIX 168, fol. 718.

[42] 3 February 1612: ASEPD, C XIX 168, fol. 707.

[43] 23 March 1612: ASEPD, C XIX 168, fols. 721–22.

[44] 24 June 1612: ASEPD, C XIX 168, fol. 750. Later, Mancini acknowledged that a tract he had written on the conclave, embellished and expanded, was circulating under somebody else’s name: the thief was a friend and they shared the same patron.

[45] 26 June 1620, transcribed in Maccherini, ‘Caravaggio e i caravaggeschi’, pp. 346–47. Steps towards the publication of the ‘Considerazioni’ were taken, but it did not materialize.

[46] On vertical transmission, see Richardson, p. 20. On competition for patronage from astronomers and mathema- ticians, see Mario Biagioli, *Galileo, Courtier: The Practice of Science in the Culture of Absolutism* (Chicago: University of Chicago Press, 1993).

[47] 29 October 1617: ASEPD, C XIX 169, fol. 546^v^. See Filippo De Vivo, *Information and Communication in* Venice: Rethinking Early Modern Politics (Oxford: Oxford University Press, 2007).

[48] 15 April 1617: ASEPD, C XIX 169, fol. 483.

[49] 10 February 1617: ASEPD, C XIX 169, fol. 463. On the use of print in relation to concerns with plagiarism and claims to priority, see Biagioli, *Galileo’s Instruments of Credit*.

[50] British Library, MS Sloane 3133; for a description, see Giuseppe Fanchiotti, *I mss italiani in Inghilterra*, 1st ser., *Londra, Il Museo Britannico*, 3 vols (Caserta: Stabilimento Tipo-Litografico Salvatore Marino, 1899–1902), i, La collezione Sloane, 137–38

[51] Mancini to Deifebo, 28 March 1620, in Maccherini, ‘Caravaggio e i caravaggeschi’, pp. 342–43

[52] Murray Jones Peter, ‘Witnesses to Medieval Medical Practice in the Harley Collection’, Electronic British Library Journal, article 8 (2008), 1–13, 〈http://www.bl.uk/eblj/2008articles/articles.html〉 [accessed 7 July 2010].

[53] On literary assemblages and their presentation as gifts, see Richardson, pp. 41–44.

[54] 3 February 1612: ASEPD, C XIX 168, fol. 707.

[55] 24 June 1616: ASEPD, C XIX 169, fol. 359^v^.

[56] 3 July 1621: ASEPD, C XIX 169, fol. 940.

[57] 16 July 1621: ASEPD, C XIX 169, fol. 944.

[58] 11 June 1621: ASEPD, C XIX 169, fol. 936.

[59] For the nature of family papers, see ASEPD, C XIX 163.

[60] 29 December 1623: ASEPD, C XIX 170, fol. 292^v^.

[61] 17 February 1624: ASEPD, C XIX 170, fol. 406.

[62] Now in BCS, C IX 3, folder 4.

[63] BAV, MSS Barb. Lat 4314–17; for a description of these manuscripts and identification of the scribes’ handwriting, see Adriana Marucchi, ‘Introduzione’, in Mancini, *Considerazioni sulla Pittura*, i, xxiii–xxvi and lvii–lix

[64] They are mostly in MS Barb. Lat. 4316.

[65] MS Barb. Lat. 4316, fols. 270–86.

[66] Mancini’s nephew to Deifebo

